# Urinary and Renal Diseases

**Published:** 1886-02

**Authors:** 


A Practical Treatise on Urinary and Renal Diseases. *Including Urinary Deposits, Illustrated by numerous Cases and Engravings. By* William Roberts, M. D., F. R. S., Etc. *Assisted by* Robert Maguire, M. D., *London. Fourth edition. Philadelphia:* Lea, Brothers & Co.

Among the numerous works on renal and urinary diseases now in circulation, perhaps Dr. Roberts' has the best claim to be regarded as "standard." The present edition shows evidence of having been carefully revised, and appears to be well up with the times. The article on "Micro-organisms in the Urine" has been expanded and improved, although it is by no means complete. The chapter on "Albuminuria" is an excellent *exposé* of the present doctrines regarding the efficient causes of albumen in the urine. In this chapter we are glad to notice that something like order is at last evolving out of the chaos of professional opinion which has so long prevailed concerning albuminuria. The author amplifies and insists upon the clinical value of his "minimetric method" of testing for albumen in the urine. "Part II." treats of "Diseases of which the chief characteristic is an alteration of the urine," and includes "Diabetes Insipidus," "Diabetes Mellitus," "Gravel and Calculus" and "Chylous Urine." The section on the treatment of "Diabetes Mellitus" is very incomplete and unsatisfactory. The author's experience with strychnia and codeia is such as to excite the suspicion that neither of these remedies has received a fair and full trial at his hands. "Part III." treats of "Organic Diseases of the Kidneys," commencing with "Congestion of the Kidneys" and ending with "Anomalies of Position, Form and Number of the Kidneys." It is this portion of the work which will more particularly interest the practical physician. It is essentially a clinical work, and as such possesses a peculiar value to the busy practitioner.

We do not like the author's clumsy classification of Bright's diseases, but on the other hand the time for an irrevocable classification of renal diseases has not yet come. Again, it seems to us that so experienced a clinician as Dr. Roberts should contribute more liberally to the field of therapeutics, but then we remember that the treatment of renal affections is still contradictory and unsatisfactory to a degree almost discouraging. These faults can therefore be forgiven. "Suppuration of the Kidney," "Pyelitis and Pyonephrosis," and the consequences arising therefrom, are treated evidently by "one who knows." We especially commend the chapter on "Entozoa in the Kidneys," as perhaps the best in any one of the common text-books. Finally, in spite of the faults we have mentioned, and the faults we have *not* mentioned, Dr. Roberts' is an eminently useful and practical book, and we congratulate the author on its deserved popularity with the profession.

